# Dapagliflozin Ameliorates Renal Tubular Ferroptosis in Diabetes via SLC40A1 Stabilization

**DOI:** 10.1155/2022/9735555

**Published:** 2022-08-10

**Authors:** Bin Huang, Wenjie Wen, Shandong Ye

**Affiliations:** ^1^Department of Endocrinology, The First Affiliated Hospital of USTC, Division of Life Science and Medicine, University of Science and Technology of China, Hefei, Anhui 230001, China; ^2^Division of Life Sciences, University of Science and Technology of China, Hefei, Anhui 230001, China

## Abstract

Tubular injury has been shown to play a critical role in the morbidity of diabetic kidney disease (DKD); ferroptosis often occurs in tubules during renal disease development. This study was aimed at evaluating the inhibitory effects and potential mechanism of dapagliflozin (DAPA) against diabetic-related ferroptosis in the kidney. C57BL/6 mice were fed a high-fat diet (HFD) for 12 weeks, administered a small dose of streptozocin (STZ) for three consecutive days by intraperitoneal injection, and then orally administered dapagliflozin (10 mg/kg/day) for 8 weeks. Mouse blood and urine samples were collected, and their renal cortices were harvested for subsequent investigations. The effects of DAPA were also evaluated in HK-2 cells subjected to simulated diabetic conditions through excess glucose or palmitic acid (PA) administration. DAPA significantly ameliorated tubular injury independently of glycemic control in diabetic model mice. In vivo and in vitro investigations showed that dapagliflozin ameliorated tubular injury by inhibiting ferroptosis. Docking experiments demonstrated that dapagliflozin and SLC40A1 could bind with each other and may consequently reduce ubiquitination degradation. In conclusion, in this study, the tubular protective effects of DAPA, irrespective of glycemic control, were observed in a diabetic mouse model. DAPA ameliorated ferroptosis during diabetic tubular injury via SLC40A1 stabilization, and this may be the mechanism underlying its action. To the best of our knowledge, this is the first study to investigate the ferroptosis inhibitory effects of DAPA in the treatment of DKD.

## 1. Introduction

Diabetic kidney disease (DKD) is a common and morbid chronic diabetic complication. Approximately 30–40% of patients with type 2 diabetes mellitus (T2DM) develop DKD, and in approximately 50% of them, the disease progresses to end-stage renal disease (ESRD) [1]. Hyperglycemia is considered to drive the development of DKD. However, existing intensive glycemic control has not reduced the prevalence of DKD, and inhibitors of the renin-angiotensin-aldosterone system (RAAS) have been proven to have renal protective function on DKD patients, but the clinical effect is not always satisfactory [2, 3]. As a whole, despite the advancements made in diabetic renal pathologies over the years, DKD still causes significant mortality and morbidity [4]. Therefore, there is an urgent need for the discovery of novel therapeutic targets or drugs for the management of DKD.

Ferroptosis, which is a novel type of programmed cell death, is involved in the processes of inflammation and oxidation in various human disease states, including DKD [5, 6]. Intracellular iron homeostasis is essential for cell survival, while iron overload contributes to ROS overproduction through the Fenton reaction, thereby facilitating ferroptosis [7]. Of note, solute carrier family 40 member 1 (SLC40A1, also known as FPN1) is the only discovered iron export protein in mammals, and inhibiting SLC40A1 induces ferroptosis [8]. The study of Hao et al. shows that diabetes decreased the expression of SLC40A1 mediating ferroptosis, which induced diabetic cognitive dysfunction [9]. Due to the special reabsorption function of renal tubular tissue (including glucose and iron), which contains numerous mitochondria, its metabolic activity and energy demand are high; diabetes induces impairments in mitochondrial energy metabolism, which results in significant intrarenal oxidative stress and cell damage [10]. Tubular injury has been shown to play a critical role in DKD progression, which correlates with renal functional deterioration, a primary change associated with the disease [11]. Ferroptosis often occurs in renal tubules during the development of renal diseases because of the sensitivity of renal tubular tissue to oxidative stress and lipid peroxidation [12]. Zhu et al. recently demonstrated that miR-4735-3p facilitates ferroptosis and tumor suppression in clear cell renal cell carcinoma by targeting SLC40A1 [13]. However, it has not been reported whether SLC40A1 is involved in renal tubular ferroptosis in the diabetic conditions.

Dapagliflozin (DAPA), one of the clinically employed hypoglycemic agents for diabetes treatment, functions primarily by decreasing glucose reabsorption in the proximal tubule via sodium-glucose cotransporter 2 (SGLT2) [14]. The cardioprotective and renoprotective function beyond their hypoglycemic effect of SGLT2 inhibitors (SGLT2i) has recently been reported and recognized [15–17]. Quagliariello et al. reported that empagliflozin reduced ferroptosis in doxorubicin-treated mice through the NLRP3 and MyD88-related pathways, which resulted in significant improvements in cardiac function [18]. However, the DKD-improving effects of SGLT2i through the inhibition of tubular ferroptosis have not been evaluated. In this study, we sought to determine whether diabetes-related ferroptosis could be inhibited by DAPA, thereby delaying the progression of DKD. This study is expected to provide a new perspective on the therapeutic mechanism of DAPA in DKD.

## 2. Materials and Methods

### 2.1. Animal Experiments

Eight-week-old specific pathogen-free C57BL/6 mice weighing 20-23 g were purchased from Shandong Kesibei Biotechnology Co., Ltd., China. All mice were maintained in a 48 ± 10% humid environment at room temperature (20 ± 1°C), under a 12 h light/dark cycle, with free access to food. All animal experiments were strictly carried out following the guidelines stated by the Ethics Committee of the First Affiliated Hospital of the University of Science and Technology of China (Anhui Provincial Hospital). Every effort was made to minimize the number of animals used and their suffering. Eight mice were randomly selected to constitute the normal control (NC) group (*n* = 8) and were fed a normal chow diet; the rest of the mice were fed a high-fat diet (HFD). With small-dose streptozocin (STZ) administration by intraperitoneal injection (50 mg/kg, dissolved in 0.1 mol/L citrate buffer, pH = 4.2, for three consecutive days) after 4 months of HFD feeding, mice in the NC group were injected an equal amount of citrate buffer. After one week, tail vein blood glucose levels were measured using a glucometer. Mice with random blood glucose levels > 16.7 mmol/L for 3 days were used for subsequent investigations. These mice were randomly divided into three groups; the T2DM group (*n* = 7), in which mice were administered sterile saline daily by gavage for 8 weeks; the DAPA group (*n* = 8), in which mice were administered DAPA (10 mg/kg/day) daily by gavage for 8 weeks; and the glibenclamide (GLIB, a clinically employed antidiabetic molecule, which has no protective effect on the kidney beyond glycemic control) group (*n* = 7), in which mice were administered GLIB (2.5 mg/kg/day) daily by gavage for 8 weeks. At the 8th week, mouse urine was collected and weighed. Then, the mice were fasted overnight, anesthetized by intraperitoneal injection of sodium pentobarbital (30 mg/kg), and sacrificed by cervical dislocation, and blood samples were collected. Mouse kidneys were also collected; one part of each kidney was fixed in 4% polyoxymethylene-phosphate buffer for histological analysis, and the other part was snap-frozen for subsequent molecular analyses.

### 2.2. Detection of Blood and Urine Indicators

When the treatment was over, blood was collected from the tail vein of the mice. Hemoglobin A1C (HbA1C) and fasting blood glucose (FBG) levels were determined using a commercial ELISA kit (Meimian, Jiangsu, China). Urine creatinine (UCR) levels were measured using the picrate method (Jiancheng, Nanjing, China). Urine albumin (ALB), Retinol-Binding Protein (RBP), Tamm Horsfall protein (THP), *α*-Microglobulin (a1MG), 8-hydroxy-2 deoxyguanosine (8OHdG), and 8-iso prostaglandin (8iso-PG) levels were measured using an ELISA kit (Meimian, Jiangsu, China). Urine ALB/UCR (UACR), PCX/UCR, RBP/UCR, THP/UCR, a1MG/UCR, 8OHdG/UCR, and 8iso-PG/UCR ratios were subsequently calculated to exclude the effect of urine concentration.

### 2.3. Electron Microscopic Observation

Mouse renal cortices were harvested and fixed using a fixing solution for the preparation of ultra-thin sections. After postfixation with 1% OsO4 and gradient dehydration, they were dehydrated in a series of ethanol (50–100%) and embedded in resin, sliced, and placed on a formvar carrier grid, followed by uranyl acetate and lead citrate treatments. Then, the sections were examined under a 20,000x transmission electron microscope (JEM1400, JEDL, Japan). Ultrastructural damage to mitochondria was assessed.

### 2.4. Quantitative PCR Analysis

Total RNA was extracted from mouse kidney tissues using the TRIzol reagent and reverse-transcribed to cDNA. Quantitative PCR was performed using SYBR Green PCR technology on a Bio-Rad CFX96 Touch Real-Time PCR System. The sequences of the primers used for real-time PCR are listed in Supplement Table S1. The PCR cycling conditions were as follows: predenaturation at 95°C for 5 min, 40 cycles of 95°C for 10 s and 60°C for 30 s, and one cycle of 95°C for 15 s, 60°C for 60 s, and 95°C for 15 s. Cycle threshold (Ct) values were determined by the comparative Ct method and normalized to *β*-actin levels.

### 2.5. Cell Culture and Treatment

Human kidney proximal tubular cells (HK-2 cells) were maintained in DMEM supplemented with 5.5 mmol/L glucose, 10% fetal bovine serum (FBS), 100 U/mL penicillin, and 100 mg/mL streptomycin and were cultured at 37°C in a 95% humid and 5% CO_2_-containing environment. BSA-conjugated PA (Sigma-Aldrich, St. Louis, MO, USA) was prepared as previously described [19]. HK2 cells were then divided into 6 groups: (1) group HG: the cells were treated with 50 mM glucose; (2) group HG+DAPA: after the cells were treated with HG for 48 h, they were treated with DAPA for 24 h; (3) group PA: the cells were treated with 300 *μ*M PA; (4) group PA+DAPA: after the cells were treated with PA for 48 h, they were treated with DAPA for 24 h; (5) group erastin: the cells were treated with erastin; and (6) group erastin +DAPA: after the cells were treated with erastin for 48 h, they were treated with DAPA for 24 h.

### 2.6. RNA Interference Analysis

HK2 cells were transfected with specific or scrambled small interfering RNAs (siRNAs) using a Lipofectamine 2000 device (Invitrogen, Grand Island, NY, USA) following the manufacturer's protocol. siRNAs were purchased from General Biol (Chuzhou, China). The sequences of the siRNAs were as follows: si-SLC40A1: 5′-CAAGAAUGCUAGACUUAAATT-3′, scramble: 5′-UUUAAGUCUAGCAUUCUUGTT-3′, si-SGLT2: 5′-GUAUGACAACAGCCUCAAGTT-3′, and scramble: 5′-UUCUCCGAACGUGUCACGUTT-3′. Unless otherwise specified, DAPA was added to the cells 24 h posttransfection.

### 2.7. Western Blot and Immunoprecipitation

For western blot, total proteins in renal tissues and cells were extracted by grinding the tissues in the RIPA buffer; protein concentrations were measured through the BCA assay. After centrifugation at 13000 g at 4°C for 10 min, the same quantity of protein was extracted with 10% sodium dodecyl sulfate-polyacrylamide gel electrophoresis (SDS-PAGE) gels and then transferred to nitrocellulose filter (NC) membranes. Then, the membranes were blocked in 5% skim milk. The proteins were detected using specific primary anti-*β*-actin (1 : 5000, Enogene, Nanjing, China), anti-SLC40A1 (1 : 1000, Bioss, Beijing, China), anti-GPX4 (1 : 1000, Bioss) and anti-SLC7A11 (1 : 1000, Bioss) antibodies, anti-TFR1 (1 : 1000, Affinity, Jiangsu, China) antibodies, anti-NCOA4 (1 : 1000, Affinity) antibodies, anti-FTH1 (1 : 1000, Bioss) antibodies, anti-SLC39A8 (1 : 1000, ABclonal, Wuhan, China) antibodies, and anti-ubiquitination(1 : 1000, PTM, Hangzhou, China). Protein bands were visualized using a Super ECL kit (UElandy, Suzhou, China) and analyzed using the ImageJ software. For immunoprecipitation, cells were lysed in MCBL buffer (50 mM Tris-HCl, 150 mM NaCl, 5 mM EDTA, and 0.5% NP-40), supplemented with protease inhibitors (Topscience, Shanghai, China). Protein A/G magnetic beads (Bimake, USA) were incubated with antibody for 1 hour. Then, cell lysates were added and incubated overnight at 4 t. The next day, immunocomplexes were washed three times using lysis buffer, resolved by SDS/PAGE, and detected by western blot.

### 2.8. Determination of ROS Generation

Intracellular ROS levels were evaluated using 2′,7′-dichlorofluorescin diacetate (DCFH-DA) (Solarbio, Beijing, China). In brief, the cells were incubated with 10 *μ*mol/L DCFH-DA at 37°C for 20 min and then washed with PBS. Fluorescence was evaluated using a fluorescence microscope at excitation and emission wavelengths of 488 and 525 nm, respectively.

### 2.9. GSH, MDA, and Iron Assays

Kidney tissue and cell samples were homogenized on ice using a homogenizer and then centrifuged for supernatant collection. Glutathione (GSH) levels were measured using a reduced GSH assay kit (Jiancheng, Nanjing, China), and optical density was measured at 405 nm. Malondialdehyde (MDA) levels were measured using a lipid peroxidation MDA assay kit (Biosharp, Hefei, China), and optical density was measured at 535 nm. Tissue iron levels were measured using a tissue iron assay kit (Jiancheng, Nanjing, China), and optical density was measured at 520 nm. Fe^2+^ concentrations were measured using a ferrous ion colorimetric assay kit (Elabscience, Wuhan, China), and optical density was measured at 590 nm. The kits were used following the manufacturers' instructions.

### 2.10. Cell Viability Assay

Cell viability was evaluated using the Cell Counting Kit-8 (Topscience, Shanghai, China), according to the manufacturer's instructions. In brief, cells were seeded in 96-well plates at a density of 3000 cells per well and exposed to various concentrations of the compounds for specified durations. Ten microliters of the working reagent was added to each well and incubated for 2 h at 37°C. Absorbance was measured at 450 nm using a microplate reader (Thermo Fisher Scientific, USA). Optical density was taken to be proportional to the number of living cells in the plate.

### 2.11. Lipid Reactive Oxygen Species Measurement

Cells were treated as indicated; then, 50 *μ*M BODIPY™ 665/676 (Thermo Fisher Scientific, USA) was added to the cells, which were incubated for 1 h. Excess BODIPY™ 665/676 was removed by washing the cells twice with PBS. Representative images were obtained using a confocal microscope.

### 2.12. Molecular Docking

The binding mode between dapagliflozin and SLC40A1 was determined using AutoDock 4.2. The three-dimensional (3D) structure of SLC40A1 was downloaded from the RCSB Protein Data Bank (PDB ID: 6 W4S), and that of dapagliflozin was obtained from the NCBI PubChem Compound (CID: 9887712) database. The AutoDockTools 1.5.6 software package was used to generate the docking input files.

### 2.13. Statistical Analysis

Statistical analyses were performed using SPSS 26.0 (IBM, Inc., Armonk, NY, USA). Data are presented as mean ± standard deviation (mean ± SD). Comparisons between two groups were performed using Student's *t*-test. One-way ANOVA with post hoc analysis of variance was used to compare data between multiple groups. For normally distributed data, pairwise comparisons were conducted using the LSD test. Otherwise, pairwise comparisons were conducted using Dunnett's T3 test. Values of *P* < 0.05 were considered statistically significant.

## 3. Results

### 3.1. The Additional Renal Protective Effect of Dapagliflozin in T2DM Mice

**Figure 1:**
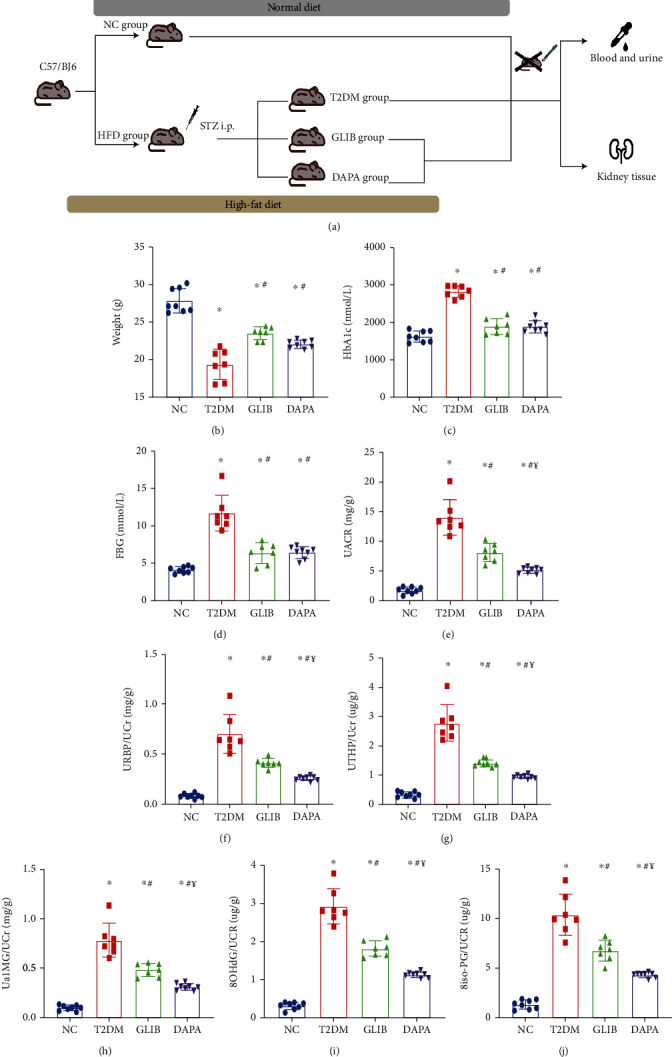
The additional renal protective effect of dapagliflozin in T2DM mice: (a) workflow of the experiment; (b) body weights in each group; (c) HbA1c concentrations in each group; (d) FBG concentrations in each group; (e) urinary ACRs; (f) urinary RBP/creatinine ratio; (g) urinary THP/creatinine ratio; (h) urinary a1MG/creatinine ratio; (i) urinary 8-OHdG/creatinine ratio; (j) urinary 8iso-PG/creatinine ratio. Values are the mean ± SD. *n* = 7 mice in the T2DM group and GLIB group, respectively; *n* = 8 mice in the NC group and DAPA group, respectively. ^∗^*P* < 0.05 vs. NC; ^#^*P* < 0.05 vs. T2DM; ^¥^*P* < 0.05 vs. GLIB.

An animal T2DM model was established by feeding male C57BL/6 mice high-fat diet (HFD)/low-dose STZ. In our experiment, we used a clinically employed antidiabetic drug (GLIB) as a hypoglycemic control, which has no protective effect on the kidney beyond glycemic control; the workflow for this experiment is shown in Figure 1(a). These mice were used to evaluate the therapeutic effects of DAPA against DKD. As shown in Figures 1(b)–1(d), there was a significant increase in HbA1c and FBG levels and a decrease in body mass in mice in the T2DM group; however, these changes were reversed when treated with DAPA or GLIB, indicating similar hypoglycemic effect was produced by those two drugs. At the end of the study, the levels of different urine proteins were evaluated (Figures 1(e)–1(j)). We found that DAPA and GLIB significantly ameliorated the increased urine protein excretion levels in T2DM mice. Furthermore, as compared to GLIB, DAPA significantly decreased UACR levels and URBP/UCR, Ua1MG/UCR, UTHP/UCR, 8OHdG/UCR, and 8iso-PG/UCR ratios, indicating that its additional renal protective effects were independent of glycemic control.

### 3.2. Dapagliflozin Ameliorates Tubular Injury in T2DM Mice Independently of Glycemic Control

**Figure 2:**
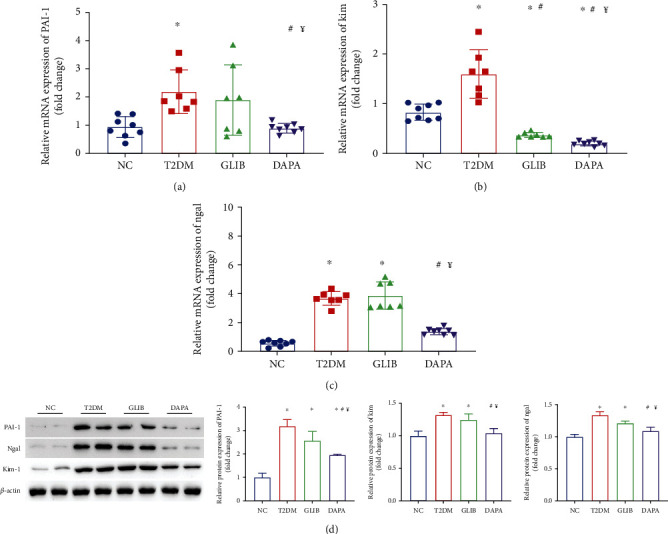
Dapagliflozin ameliorates tubular injury in T2DM mice independently of glycemic control. RT-PCR results of PAI-1 (a), KIM1 (b), and Ngal (c) in the kidney. (d) Quantification of the average band densities calculated from different western blots and the protein levels of PAI-1, KIM1, and Ngal in the kidney tissue in these groups. *n* = 7 mice in the T2DM group and GLIB group, respectively; *n* = 8 mice in the NC group and DAPA group, respectively. ^∗^*P* < 0.05 vs. NC; ^#^*P* < 0.05 vs. T2DM; ^¥^*P* < 0.05 vs. GLIB.

Considering those urine biomarkers were reflecting tubular injury, we evaluated the renal mRNA levels of Kim, Ngal, and PAI-1, all of which were typical markers of tubular damage [20]. We found that Kim, Ngal, and PAI-1 expression was higher in the kidneys of T2DM mice than in those of mice in the NC group; however, as shown in Figures 2(a)–2(c), their expression was significantly reduced following dapagliflozin administration when compared to the GLIB group. The protein level had also validated those phenomena (Figure 2(d)). Collectively, these findings indicate that dapagliflozin treatment might significantly ameliorate tubular injury in T2DM mice independently of glycemic control.

### 3.3. Dapagliflozin Ameliorated Ferroptosis in T2DM Mice

**Figure 3:**
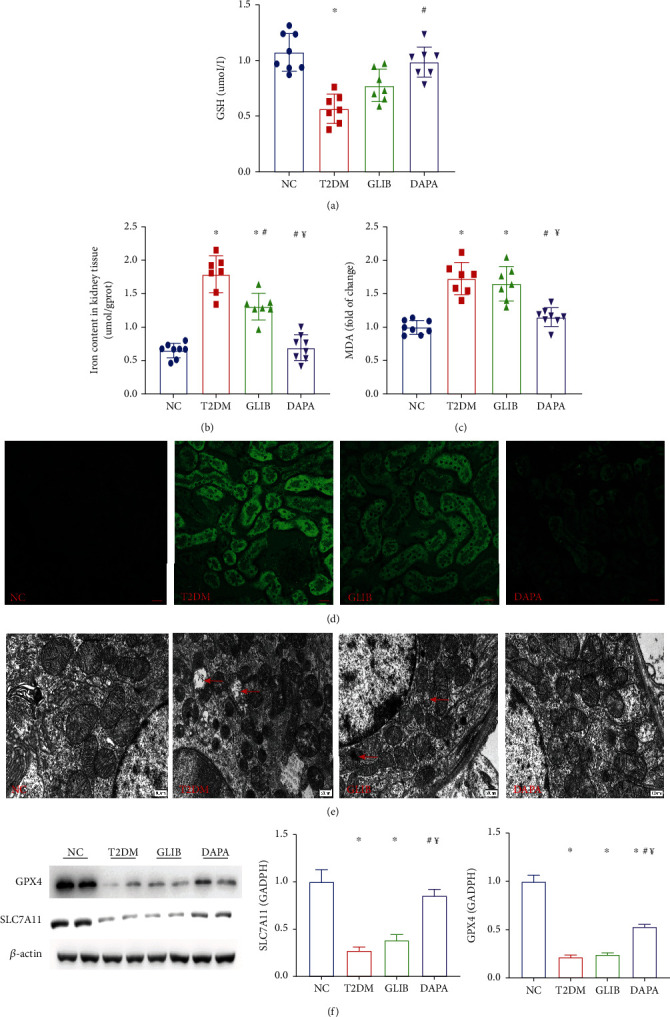
Dapagliflozin ameliorated ferroptosis in T2DM mice. (a) GSH, (b) iron, and (c) MDA contents in the kidneys of mice in these groups. (d) The production of ROS (green) in each group. (e) Transmission electron microscopy was used to detect the mitochondrial morphology of renal tubular epithelial cells in each group. The red arrow indicates the damaged mitochondria (ruptured mitochondrial membranes and the disappearance of mitochondrial cristae). (f) Quantification of the average band densities calculated from different western blots and the protein levels of GPX4 and SLC7A11 in the kidney tissue in these groups. Values are the mean ± SD. *n* = 7 mice in the T2DM group and GLIB group, respectively; *n* = 8 mice in the NC group and DAPA group, respectively. ^∗^*P* < 0.05 vs. NC; ^#^*P* < 0.05 vs. T2DM; ^¥^*P* < 0.05 vs. GLIB.

Ferroptosis is an iron-dependent and regulated type of necrosis characterized by increased lipid peroxidation [21]. Thus, to determine the involvement of ferroptosis in DKD, the levels of glutathione, iron ions, lipid peroxidation, and massive reactive oxygen species (ROS), as well as the expression levels of some ferroptosis markers, were determined in kidney tissues. GSH, iron ion, and MDA levels in mouse kidney tissues were found to be significantly higher in the T2DM group than in the control group; however, these abnormal levels were significantly restored after 8 weeks of dapagliflozin administration (Figures 3(a)–3(c)). Furthermore, the kidney tissues were stained with 2′,7′-dichlorofluorescein diacetate to evaluate ROS generation. Minimal background fluorescence was observed in the kidney tissues of mice in the NC group; the kidney tissues of mice in the diabetic group showed the highest fluorescence intensities, and these significantly decreased following dapagliflozin administration (Figure 3(d)). In line with this finding, transmission electron microscopy also revealed ruptured mitochondrial membranes and the disappearance of mitochondrial cristae in the kidney cells of mice in the T2DM and glibenclamide groups; these changes were significantly ameliorated by treatment with dapagliflozin (Figure 3(e)). GPX4 and SLC7A11 are considered to be the primary proteins for ferroptosis prevention, and the deletion or inhibition of GPX4/SLC7A11 could induce ferroptosis. In this study, there was a significant decrease in GPX4 and SLC7A11 levels in mice in the T2DM and GLIB groups; however, their expression levels were upregulated in mice in the DAPA treatment group (Figure 3(f)). Collectively, these findings suggested that dapagliflozin may ameliorate tubular injury in the T2DM mouse model by inhibiting ferroptosis.

### 3.4. Dapagliflozin Ameliorates Ferroptosis in HK-2 Cell Injury Models In Vitro

**Figure 4:**
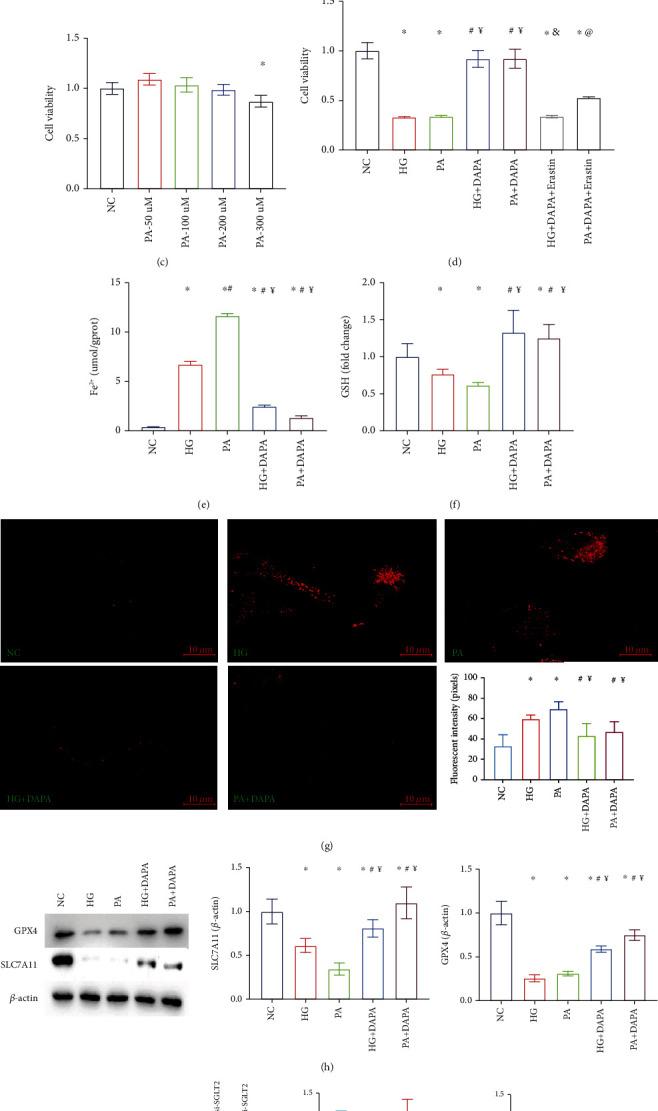
Dapagliflozin ameliorates ferroptosis in HK-2 cell injury models in vitro. (a) The cell viability assay revealed a significant dose-dependent increase cell death in erastin-stimulated cells as compared to control cells; CCK-8 assays with different stimulating glucose (b) or PA (c) concentrations in HK-2 cells. (d) Dapagliflozin inhibited HG- or PA-induced cell death in HK-2 cells, and this effect was reversed by erastin; (e) iron ion, (f) GSH, and (g) MDA contents in these groups. (h) Quantification of the average band densities calculated from different western blots and the protein levels of GPX4 and SLC7A11 in HK-2 cells in these groups. (i) SGLT2-siRNA was validated by western blot and the expression of SLC7A11 and GPX4 in those groups. Values are the mean ± SD. *n* = 3 independent experiments. ^∗^*P* < 0.05 vs. NC; ^#^*P* < 0.05 vs. HG or erastin 1 *μ*M group; ^¥^*P* < 0.05 vs. PA; ^&^*P* < 0.05 vs. HG+DAPA; ^@^*P* < 0.05 vs. PA+DAPA.

To describe cell death via ferroptosis in kidney cells, we analyzed HK-2 cells treated with various concentrations of erastin. The CCK-8 assay revealed a significant dose-dependent increase cell death in erastin-stimulated cells as compared to control cells (*P* < 0.05 for 2 *μ*M; *P* < 0.01 for 4 and 6 *μ*M; and *P* < 0.001 for 8 and 10 *μ*M; Figure 4(a)), suggesting that HK-2 cells were sensitive to ferroptosis. Diabetes has a complex pathophysiological mechanism, which involves changes in glucose and lipid metabolism. To verify the previously obtained findings, we pretreated HK-2 cells with different concentrations of high glucose (HG) and palmitic acid (PA) for 48 h to model the metabolic disorder environment observed during diabetes. The cell viability assay showed that as compared to the NC group, cell death significantly increased when glucose concentrations exceeded 50 mM or at a PA concentration of 300 *μ*M (Figures 4(b) and 4(c)). Next, we assessed, in vitro, whether the tubular injury-ameliorating effects of dapagliflozin were dependent on ferroptosis signaling. The CCK-8 assay showed that dapagliflozin inhibited high glucose- (50 mM) or PA- (300 *μ*M) induced cell death in HK-2 cells, and this effect was reversed by erastin (2 *μ*M) (Figure 4(d)). We further evaluated ferroptosis-related markers in these groups. After treatment with 50 mM glucose or 300 *μ*M PA, iron ion (Figure 4(e)) and glutathione levels (Figure 4(f)), as well as MDA content (Figure 4(g)), increased; however, their levels significantly decreased following dapagliflozin administration. The expression changes of GPX4 and SLC7A11 levels in vitro were also consistent with the mouse model (Figure 4(h)). Collectively, these findings suggested that dapagliflozin ameliorates ferroptosis in HK-2 cell injury models. The hypoglycemic function of dapagliflozin is primarily by decreasing glucose reabsorption in the proximal tubule via SGLT2 inhibition [22]. In order to explore whether its therapeutic effect of ferroptosis also depends on SGLT2 suppression, we knocked out SGLT2 in HK-2 cells, and this was validated by western blot. There was no significant improvement in GPX4 and SLC7A11 expression when compared to the HG/PA group, indicating that the ferroptosis improvement effect of dapagliflozin was independent of inhibition of SGLT2 (Figure 4(i)).

### 3.5. Dapagliflozin Ameliorates Ferroptosis in HK-2 Cells via SLC40A1

**Figure 5:**
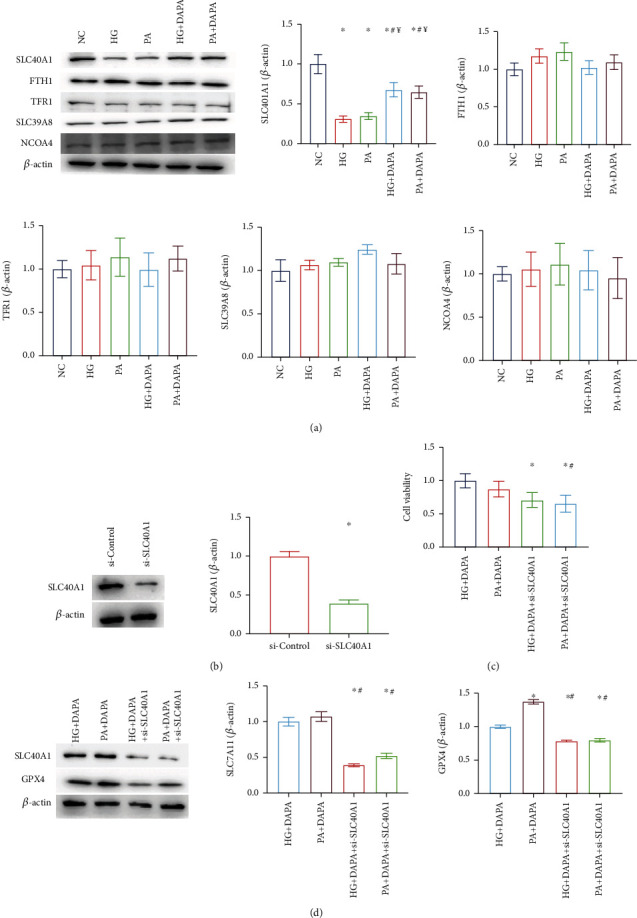
Dapagliflozin ameliorates ferroptosis in HK-2 cells via SLC40A1. (a) Fe^2+^ transport-related proteins were detected. (b) SLA40A1-siRNA was validated by western blot. (c) CCK-8 assays with these groups. (d) The expression of GPX4 and SLC7A11 in these groups. Values are the mean ± SD. *n* = 3 independent experiments. ^∗^*P* < 0.05 vs. NC; ^#^*P* < 0.05 vs. HG; ^¥^*P* < 0.05 vs. PA.

Given the important role played by iron overload in ferroptosis, a disruption in cellular iron homeostasis can contribute to tubular injury [23]. To examine the underlying mechanism governing the aberrant elevation of Fe^2+^ in HK-2 cells, the Fe^2+^ transport-related proteins were detected firstly. The protein level of SLC40A1, which is responsible for Fe^2+^ export from the cytoplasm to extracellular space, was significantly inhibited by HG or PA exposure, while DAPA administration can recover the expression of SLC40A1. However, the expression of iron metabolism-related proteins [24], including FTH1, TFR1, SLC39A8, and NCOA4, which transport cytoplasm Fe^2+^ into the lysosome for storage or mediate iron import into cells were not affected by HG or PA incubation. That is, HG/PA could inhibit intracellular iron efflux but did not affect iron influx, storage, and metabolism (Figure 5(a)). To determine the role played by SLC40A1 in diabetic tubular ferroptosis, we modified SLC40A1 levels in HK-2 cells by transfecting them with SLA40A1-siRNA, and this was validated by western blot (Figure 5(b)). Following si-SLC40A1 pretreatment, the CCK-8 assay showed that the dapagliflozin inhibited HG- or PA-induced cell death in HK-2 cells, and this effect was reversed by SLC40A1 knockout (Figure 5(c)). Similarly, there was a reversal in the therapeutic effects of dapagliflozin in the HK-2 cell injury models on GPX4 and SLC7A11 expression levels (Figure 5(d)). Those findings indicate that DAPA ameliorates ferroptosis in HK-2 cells dependent on SLC40A1 expression.

### 3.6. The Potential Mechanism of Dapagliflozin Stabilizes SLC40A1 of the Renal Tubule in Diabetes

**Figure 6:**
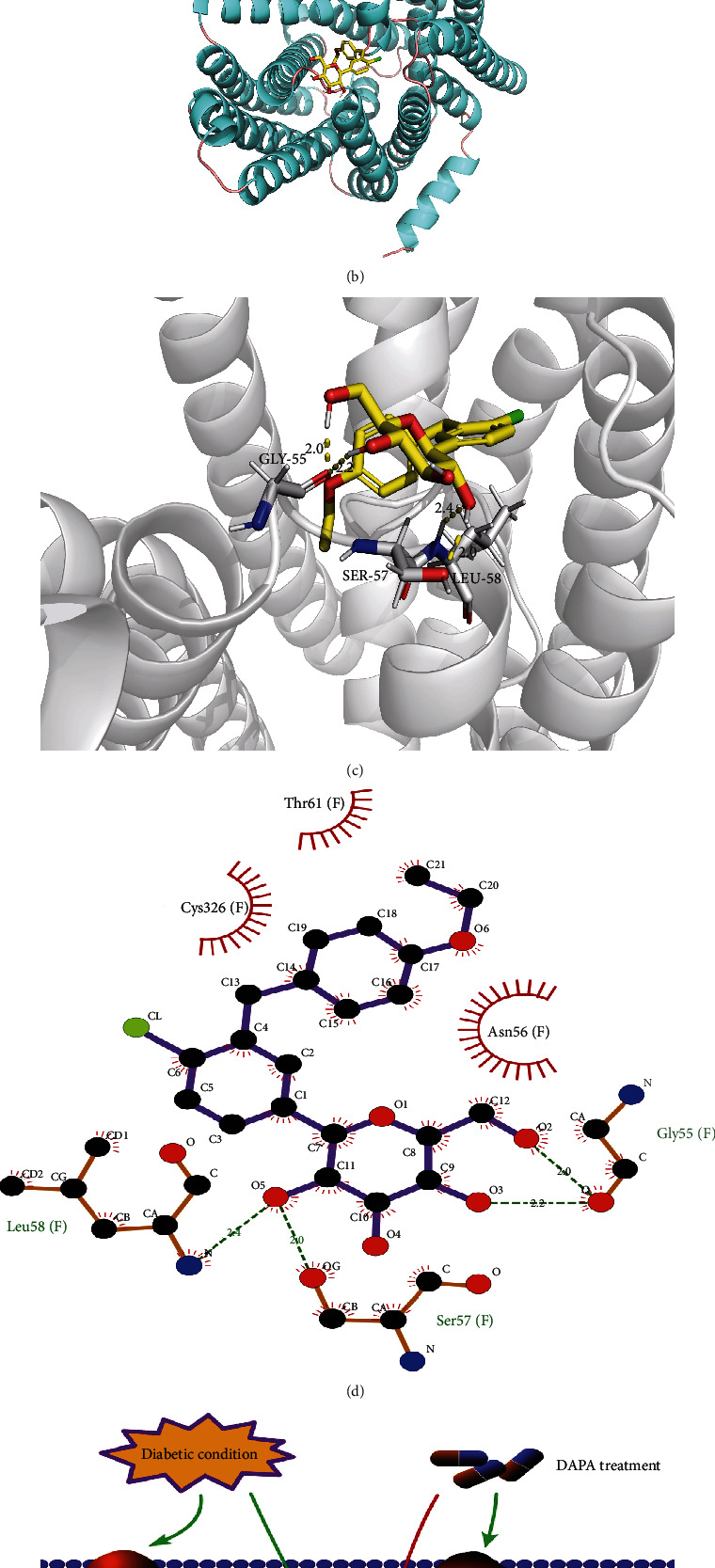
The potential mechanism of dapagliflozin stabilizes SLC40A1 of the renal tubule in diabetes. (a) The ubiquitination of SLC40A1, assessed by western blot analysis following immunoprecipitation. (b) Frontal view of the 3D model of the binding of dapagliflozin to the SLC40A1 complex. (c) Stereoview of the binding mode of dapagliflozin in its complex with SLC40A1, in which the H-bonds are depicted as yellow dotted lines. (d) Specific view of the 2D ligand interaction of dapagliflozin with SLC40A1. (e) Schematic representation of the pathways involved in DKD through the dapagliflozin-regulated SLC40A1/ferroptosis pathway.

The main function of ubiquitination is the control of protein degradation [25]. In our study, we found that HG or PA administration in HK-2 cells significantly increased ubiquitination of SLC40A1 but reduced upon DAPA treatment (Figure 6(a)). Those findings indicate that dapagliflozin ameliorates ferroptosis in HK-2 cells by SLC40A1 stabilization. To explore the interaction between dapagliflozin and SLC40A1, a molecular docking experiment was performed using AutoDock4.2. The structure of human SLC40A1 was obtained from the Protein Data Bank (PDB 6W4S). The binding affinity score for dapagliflozin to SLC40A1 was -4.31 kcal/mol. Three-dimensional ribbon models for the DAPA-SLC40A1 complex is depicted in Figure 6(b). We found that dapagliflozin has multiple hydroxyl groups and forms eight hydrogen bonds with the Gly55, Ser57, and Leu58 residues in SLC40A1. The donor-acceptor distance was in the 2.0 to 2.4 Å range (Figures 6(c) and 6(d)). Moreover, the dapagliflozin displayed hydrophobic interactions with amino acid residues Asn56, Thr61, and Cys326 of SLC40A1. The above results showed a strong and most favorable binding interaction between protein SLC40A1 and DAPA and may induce conformational changes in SLC40A1, which stabilizes SLC7A11 by reducing its ubiquitination levels.

## 4. Discussion

Previous studies have shown that dapagliflozin exerts protective effects against DKD development [26]. In this study, we observed the in vivo tubular protective effects of dapagliflozin irrespective of glycemic control. Furthermore, through animal experiments and using tubular injury cell models induced by high glucose and palmitic acid concentrations, it was confirmed that dapagliflozin and SLC40A1 could bind with each other; this may induce conformational changes in SLC40A1, which subsequently causes a reduction in ubiquitination degradation and consequently ameliorates tubular ferroptosis in diabetes (Figure 6(e)). To the best of our knowledge, this is the first study to investigate the ferroptosis inhibitory effect of dapagliflozin against DKD.

DKD, which is a major microvascular diabetic complication, is a public health problem that affects millions of people worldwide. As the disease progresses, significant proteinuria occurs in most patients and it eventually develops into chronic renal failure with uremia [27]. Despite the implementation of strict measures aimed at improving glucose and lipid metabolism and normalizing blood pressure, the risk of developing DKD has remained steady over the years [28]. As opposed to other diabetic complications, the prevalence of DKD has not significantly changed over the last 30 years [29]. At present, the renoprotective effects of a new class of antidiabetic agents known as sodium-glucose cotransporter 2 inhibitors have been demonstrated. SGLT2 inhibitors reduce the risk of dialysis, transplantation, and death due to kidney disease in individuals with type 2 diabetes and provide protection against acute kidney injury [30]. A systematic review and meta-analysis that included a total of 38 723 participants demonstrated the renoprotective effects of an SGLT2 inhibitor in T2DM patients with eGFRs ranging from 30 to 45 mL/min/1.73 m^2^ [31]. Aside from decreasing blood glucose levels, SGLT2 inhibitors also affect several other pathogenic pathways that underlie DKD [32]. In our experiment, we used a clinically employed antidiabetic drug (glibenclamide) as a hypoglycemic control, which has no protective effect on the kidney beyond its hypoglycemic effect. We observed that as compared to the glibenclamide group, dapagliflozin significantly decreased a series of typical markers of tubular injury, implying that treatment with dapagliflozin may significantly ameliorate tubular damage independently of glycemic control.

Under diabetic conditions, high glucose concentrations, advanced glycation end products (AGEs), protein oxidation products, urinary proteins, and other endogenous nephrotoxins can induce the activation, transdifferentiation, hypertrophy, and apoptosis of renal tubular epithelial cells (TECs) [33]. Recent studies have shown that TEC injury may be a primary pivotal trigger for DKD development [34]. To perform their reabsorption functions, TECs require large amounts of energy; diabetes induces impaired mitochondrial energy metabolism, which results in significant intrarenal oxidative stress and TEC damage [35]. Decreased antioxidant capacity, iron overload, and lipid peroxidation product accumulation are characteristic indicators of ferroptosis, and these were observed in the DKD models, especially in the diabetic tubular injury models [36]. Li et al. showed that ferroptosis is involved in the development of DKD and that fenofibrate-induced Nrf2 upregulation inhibits diabetes-related ferroptosis, thereby delaying DKD progression [37]. As compared to the control group, SLC7A11 and GPX4 expression was found to be significantly lower in cultured tubular epithelial cells exposed to TGF-*β*1, as well as in the kidney tissues of diabetic mice; these changes were alleviated by Fer-1 treatment, indicating that ferroptosis is involved in kidney tubular cell death under diabetic conditions [38]. Feng et al. reported that ferroptosis may promote DKD and renal tubular damage in diabetic models through the HIF-1a/HO-1 pathway [39]. A recent study by Quagliariello et al. showed that empagliflozin reduces ferroptosis in doxorubicin-treated mice through the NLRP3 and MyD88-related pathways, thereby significantly improving cardiac function [12]. However, the involvement of the ferroptosis signaling pathway in the tubular injury-ameliorating mechanism of dapagliflozin under diabetic conditions has not been determined. Thus, to determine the involvement of ferroptosis in DKD, the levels of glutathione, iron ions, lipid peroxidation, massive ROS, and ECM and the expression of some ferroptosis markers were further evaluated. Our findings suggested that dapagliflozin ameliorated tubular injury in vivo and in vitro through ferroptosis inhibition. The hypoglycemic function of DAPA is primarily by decreasing glucose reabsorption in the proximal tubule via SGLT2 inhibition. In order to explore whether its therapeutic effect of ferroptosis also depends on SGLT2 suppression, we knocked out SGLT2 in HK-2 cells; surprisingly, the inhibition effect of DAPA on ferroptosis was not simulated when compared to the HG/PA group, indicating that the ferroptosis improvement effect of dapagliflozin was independent of inhibition of SGLT2.

Iron is an essential mineral that is required for various metabolic and physiological functions in living organisms [40]. Iron-dependent cell death, generally known as ferroptosis, is an iron metabolic disorder [41]. Thus, an imbalance in iron homeostasis is associated with several pathological processes [42]. Over the last three decades, iron homeostatic disorders and iron-mediated cell toxicity have been recognized as causes and consequences of kidney injury [43]. Under diabetic conditions, extensive inflammation and/or oxidative stress may occur; these mechanisms among others may cause excessive iron retention in the kidney tubules and consequently cause iron-induced kidney injury [44]. Mitigating iron overload is a primary focus for potential therapeutic interventions for DKD management [45]. SLC40A1 (also known as ferroportin) can export iron into interstitial fluid and the general circulation through the basolateral membrane of renal tubular epithelial cells [46]. SLC40A1 downregulation compromises intracellular iron output, causing iron overload and accelerating cellular ferroptosis [47]. In this study, we observed that the HG- or PA-induced decrease in HK-2 cell SLC40A1 levels was reversed following dapagliflozin administration. Moreover, after pretreatment with si-SLC40A1, the in vitro therapeutic effects of dapagliflozin in HK-2 cell injury models were reversed. Those findings indicate that DAPA ameliorates ferroptosis in HK-2 cells dependent on SLC40A1. The main function of ubiquitination is the control of protein degradation. Many studies have shown that the ubiquitin system is involved in SLC40A1 degradation, resulting in cellular iron overload [48–50]. In this study, we found that HG or PA administration in HK-2 cells significantly increased ubiquitination of SLC40A1 but reduced upon DAPA treatment, which demonstrated that DAPA ameliorates tubular ferroptosis by SLC40A1 stabilization. To explain the potential mechanism of dapagliflozin stabilizing SLC40A1 of the renal tubule in diabetes, a molecular docking experiment was performed. The analyses showed that dapagliflozin and SLC40A1 could bind with each other; this may cause a conformational change in SLC40A1, consequently leading to a decrease in ubiquitination degradation.

Our study has some limitations. First, considering the involvement of multiple organs in iron metabolism and the complexity of the diabetic environment, a lack of tubular-targeted knockout mice constituted a limitation to this study. Second, we verified decreased ubiquitination SLC40A1 levels following treatment with dapagliflozin but did not explore the specific mechanism underlying this regulatory process. Hence, further investigations are needed to improve our understanding of how conformational changes occur in SLC40A1 and which ubiquitin enzymes are involved in the mechanism of action of dapagliflozin.

## 5. Conclusions

This study concludes that the ferroptosis pathway was involved in the tubule protective effect of dapagliflozin in diabetes, and the decrease in ubiquitination degradation of SLC40A1 after binding with dapagliflozin may be the mechanism underlying its action. To the best of our knowledge, this is the first study to investigate the ferroptosis inhibitory effects of dapagliflozin in the treatment of DKD.

## Data Availability

The datasets analyzed during the current study are available from the corresponding author.

## Supplementary Materials

Supplementary Materials The sequences of the primers used for real-time PCR are listed in Supplement Table S1.
