# Supplementary material for: Dapagliflozin Ameliorates Renal Tubular Ferroptosis in Diabetes via SLC40A1 Stabilization
Source: Oxid Med Cell Longev. 2022 Aug 10;2022:9735555. doi: 10.1155/2022/9735555 (PMC9385361; doi:10.1155/2022/9735555)
Supplement: Supplementary Materials — The sequences of the primers used for real-time PCR are listed in Supplement Table S1. [file 9735555.f1.docx]

**Table S1. The sequences of mice primers for RT-PCR analysis**

| Gene name | Primer sequence (5’-3’) | Accession No. |
| --- | --- | --- |
| β-actin | F: CATTGCTGACAGGATGCAGAAGG  R: TGCTGGAAGGTGGACAGTGAGG | NM_007393 |
| KIM1 | F: CTGGAATGGCACTGTGACATCC  R: GCAGATGCCAACATAGAAGCCC | NM_134248 |
| Ngal | F: ATGTCACCTCCATCCTGGTCAG  R: GCCACTTGCACATTGTAGCTCTG | NM_008491 |
| PAI-1 | F: CCTCTTCCACAAGTCTGATGGC  R: GCAGTTCCACAACGTCATACTCG | NM_008871 |

Accession numbers are from the GenBank database.
